# Preoperative neutrophil/lymphocyte ratio and prognostic nutritional index predict survival in patients with non-small cell lung cancer

**DOI:** 10.1186/s12957-015-0710-7

**Published:** 2015-09-30

**Authors:** Katsuhiko Shimizu, Riki Okita, Shinsuke Saisho, Ai Maeda, Yuji Nojima, Masao Nakata

**Affiliations:** Department of General Thoracic Surgery, Kawasaki Medical School, 577 Matsushima, Kurashiki, Okayama 701-0192 Japan

**Keywords:** Non-small cell lung cancer, Neutrophil/lymphocyte ratio (NLR), Prognostic nutritional index (PNI)

## Abstract

**Background:**

The immunological status, consisting of “inflammation status” and “nutritional condition,” is important for the survival of patients with various cancers, including non-small cell lung cancer (NSCLC). The neutrophil/lymphocyte ratio (NLR) reflects the inflammation status, and the prognostic nutritional index (PNI) reflects the immunological nutritional condition. In the present study, the correlation between the NLR and the PNI as well as the consistency and magnitude of the prognostic impact of the NLR and the PNI were investigated.

**Methods:**

We conducted a retrospective review of data from 334 patients who had undergone a curative resection for NSCLC. The NLR and the PNI were calculated, which was routinely performed before surgery. The correlations between the NLR and the PNI and survival were then evaluated.

**Results:**

A clear inverse correlation was observed between the NLR and the PNI. The NLR was associated with sex, smoking history, the CEA level, tumor size, and vascular invasion. The PNI was associated with sex, age, smoking history, tumor size, histological type, tumor differentiation, and vascular invasion. Patients with NLR ≥2.5 had a significantly poorer survival outcome, and patients with PNI <50 had a significantly poorer survival outcome. A multivariate analysis demonstrated that age, nodal metastasis, tumor differentiation, NLR, and PNI were independent predictors of disease-free and overall survival.

**Conclusions:**

Our study demonstrated a significant inverse correlation between the NLR and the PNI, and a high NLR and a low PNI were significantly associated with a poor survival among patients who had undergone a complete resection for NSCLC.

## Background

Lung cancer is a major cause of death in many developed countries. Surgical resection continues to play an important role in the treatment of this disease, especially during the early stages of non-small cell lung cancer (NSCLC). Over the past few decades, a number of prognostic factors for NSCLC patients following resection have been investigated. Until now, the anatomic extent of the tumor (TNM classification) has been regarded as the most powerful tool for predicting patient prognosis [1]. On the other hand, several biomarkers have been reported as predictors of survival and recurrence: (1) clinical factors (e.g., sex, age, or performance status); (2) pathological factors (e.g., histological subtype, cell differentiation, or visceral pleural invasion; and (3) many biological factors involved in cancer development and progression [2, 3].

Recently, several investigators have reported that the immunological status, consisting of the “inflammation status” and the “nutritional condition,” is important for the survival of patients with various cancers, including NSCLC. First, increasing evidence regarding the “inflammation status” has shown that the systematic inflammatory response has prognostic value for patients with various cancers [4, 5]. In particular, the neutrophil/lymphocyte ratio (NLR) has been recognized as a predictor of a poor prognosis. For NSCLC, several reports have described evidence of the prognostic value of the NLR [6–12]. Second, evidence regarding the impact of the “nutritional condition” has also been increasing. Above all, the prognostic nutritional index (PNI), which is calculated by combining the serum albumin concentration with the total peripheral blood lymphocyte count, was initially used to assess the immune-nutritional status of patients receiving gastrointestinal surgery [13]. Several reports have been shown that the PNI is a prognostic marker in patients with various cancers, including cancer of the esophagus, stomach, colorectal, pancreas, and malignant pleural mesothelioma [14–18]. Moreover, the PNI can predict the prognosis of patients with cancer regardless of the site of origin [19]. However few studies examining the PNI in patients with NSCLC have been performed [20].

To our knowledge, the clinical impacts of both the NLR and the PNI have not yet been investigated simultaneously. In the present study, we investigated the correlation between the NLR and the PNI as well as the consistency and magnitude of the prognostic impact of the NLR and the PNI among patients who had undergone a complete resection for NSCLC.

## Methods

### Patient population

We conducted this retrospective study in a total of 334 patients with NSCLC who underwent surgery at the Kawasaki Medical School Hospital between 2007 and 2012. All the patients included in the analysis met the following criteria: (1) curative resection (segmentectomy or lobectomy) with lymph node dissection; (2) neither radiotherapy nor chemotherapy administered prior to the surgery; and (3) preoperative NLR and PNI obtained before surgery. The histological diagnosis of the tumors was based on the criteria of the World Health Organization, and the TNM stage was determined according to the criteria established in 2009. This study was conducted with the approval of the institutional Ethics Committee of Kawasaki Medical School (No. 1803: approved on May 12, 2014).

### NLR and PNI evaluation

The NLR and the PNI were calculated using data from a complete blood count that was routinely performed before surgery. The receiver operating characteristic (ROC) curves identified a NLR cutoff value of 2.5 for predicting recurrence in patients (area under the curve (AUC) = 0.63, 95 % CI: 0.56–0.69, *P* < 0.001). In addition, based on a previous study, the data were dichotomized using a NLR cutoff value of 2.5 [10]. The PNI was calculated as 10 × serum albumin (g/dL) + 0.005 × total lymphocyte count (per millimeter) [13]. The PNI value of at least 50 was defined as normal, while less than 50 was regarded as mild malnutrition, less than 45 was regarded as moderate to severe malnutrition, and less than 40 was regarded as serious malnutrition [15]. The cutoff value of the PNI for clinically significant malnutrition was set at below 50 in this study.

### Follow-up

The follow-up examination schedule was arranged on an individual basis; most of the patients received medical check-ups and chest x-ray films or CT scans at least twice per year. The last follow-up review was performed on June 30, 2014. The median follow-up duration for the detection of disease-free survival (DFS) or overall survival (OS) was 32.0 months (range 3–72 months).

### Statistical analysis

All the statistical analyses were performed using the SPSS statistical package (version 17.0; SPSS, Chicago, IL). Categorical data were examined using the *χ*^2^ test. ROC curves of the NLR and PNI for the prediction of DFS or OS were generated to determine the cutoff value that yielded an optimal sensitivity and specificity. The prognostic evaluation was performed by considering the OS and DFS, which was defined as the time until lung cancer recurrence, the occurrence of a second cancer, or non-lung cancer-related death. The survival curves were estimated using the Kaplan-Meier method, and differences among the curves were evaluated using the log-rank test. Univariate and multivariate analyses were performed using the Cox proportional hazards model. Two-sided *P* values of less than 0.05 were considered statistically significant.

## Results

### Patient characteristics

The patients ranged in age from 46 to 88 years (mean, 69.3 years). There were 219 men and 115 women. The majority of patients (231, 69.1 %) had adenocarcinoma, while 69 (20.7 %) had squamous cell carcinoma, 22 (6.6 %) had large cell carcinoma, and 12 (3.6 %) had other histological types. Pathological N0 disease was confirmed in 270 patients (80.8 %), and N1 or N2 disease was confirmed in 64 patients (19.2 %). Pathological stage I disease was confirmed in 239 patients (71.5 %), and stage II or stage III disease was confirmed in 95 patients (28.5 %).

### Association between NLR/PNI value and clinicopathological findings

**Fig. 1:**
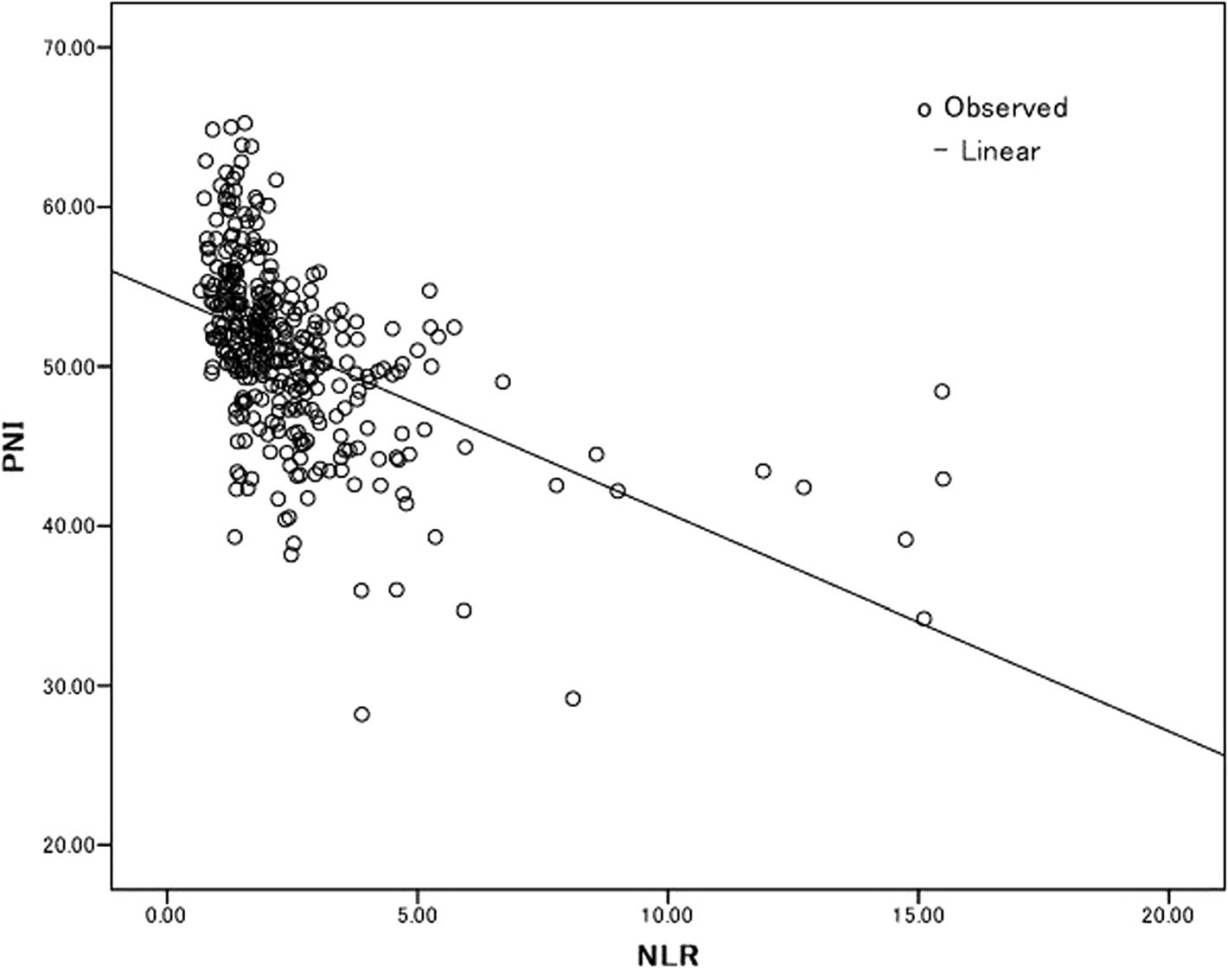
Correlation between the NLR and the PNI (*r* = −0.490, *P* < 0.001)

The NLR values of the patients ranged from 0.67 to 15.49 (mean, 2.52; median, 1.97). The mean NLR was not associated with age, smoking history, histological type, tumor differentiation, or pathological stage. On the other hand, the PNI values of the patients ranged from 28.20 to 65.25 (mean, 51.0; median, 50.80). The mean PNI was significantly lower among patients who were older, former and current smokers, and those with squamous cell carcinoma (Table 1). A significant inverse correlation was observed between the NLR and PNI values (*r* = −0.490, *P* < 0.001) (Fig. 1).

**Table 1:** Association between NLR/PNI value and clinicopathological findings

	Number	NLR	PNI
All cases	334	2.52 ± 2.06	51.03 ± 5.72
Age			
≦70	165	2.34 ± 1.91	52.20 ± 5.38*
>70	169	2.69 ± 2.18	49.59 ± 5.70*
Smoking history			
Never	122	2.27 ± 1.69	52.32 ± 5.00**^,^ ***
Former	116	2.69 ± 2.41	49.70 ± 5.65**
Current	95	2.64 ± 1.99	50.97 ± 6.36***
Histology			
Adenocarcinoma	231	2.43 + 2.07	51.87 + 5.17****
Squamous cell carcinoma	69	2.80 + 2.30	48.22 + 6.49****
Large cell carcinoma	22	2.84 + 1.42	49.55 + 6.38
Adenosquamous carcinoma	6	2.01 + 0.61	52.49 + 2.39
Pleomorphic carcinoma	6	1.90 + 0.93	55.07 + 6.92
Tumor differentiation			
Well	118	2.39 + 2.12	51.82 + 4.70
Moderate	115	2.55 + 2.29	50.96 + 6.12
Poor	101	2.59 + 1.70	50.23 + 6.30
Pathological stage			
IA	144	2.40 + 2.13	51.74 + 5.33
IB	95	2.54 + 1.68	50.30 + 6.00
IIA+IIB	55	2.60 + 2.21	50.30 + 5.50
IIIA+IIIB	40	2.75 + 2.37	51.18 + 6.58

*NLR* neutrophil/lymphocyte ratio, *PNI* prognostic nutritional index

*^,^ **^,^ ****p < 0.01

***p < 0.05

### Clinicopathological characteristics grouped according to NLR and PNI statuses

**Fig. 2:**
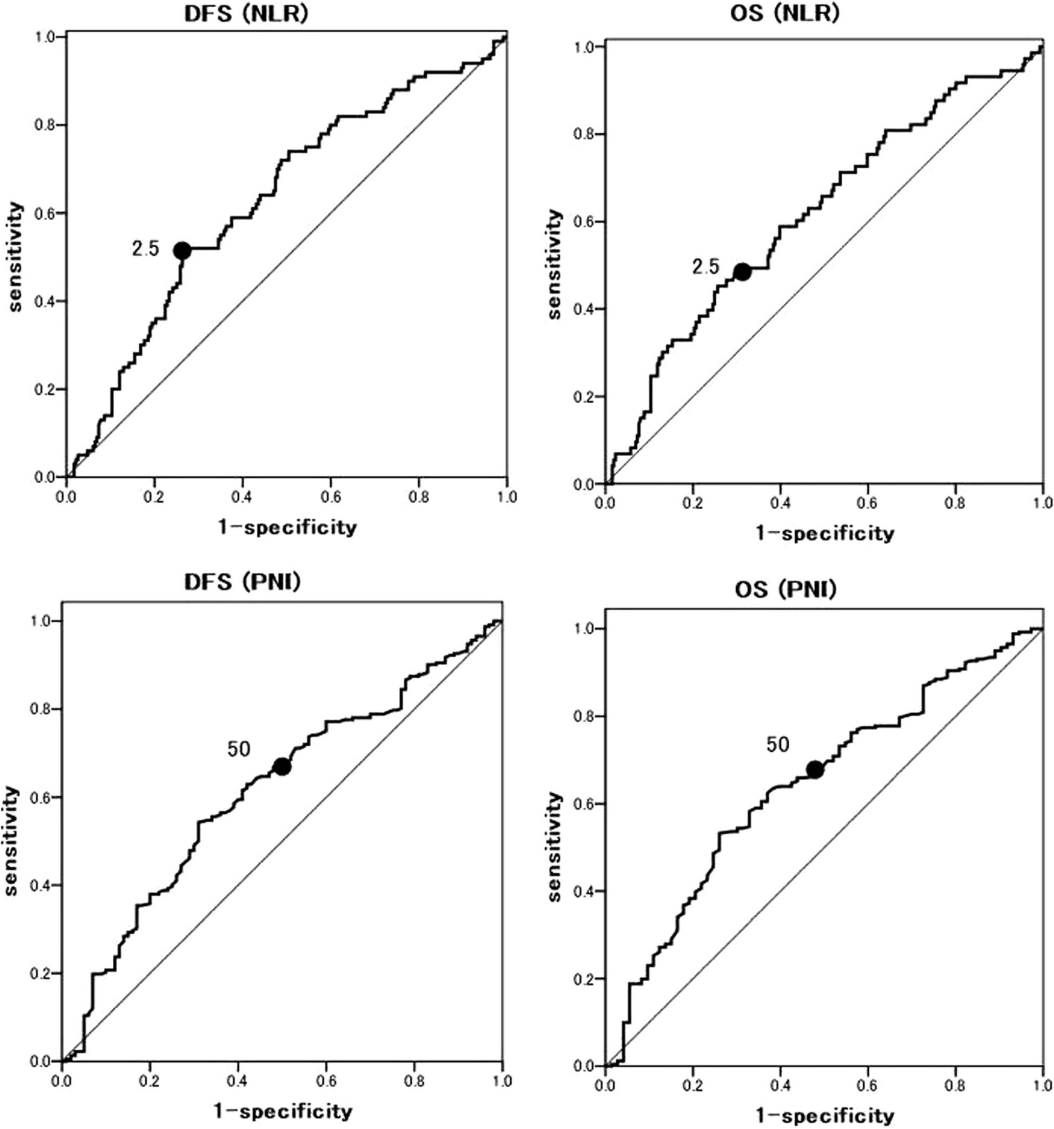
Receiver operating characteristics (ROC) curve for predicting disease-free survival (DFS) or overall survival (OS). DFS-NLR: AUC 0.63(95 % CI 0.56–0.69) *P* = 0.001. OS-NLR: AUC 0.62 (95 % CI 0.54–0.69) *P* = 0.002. DFS-PNI: AUC 0.62 (95 % CI 0.55–0.68) *P* = 0.001. OS-PNI: AUC 0.64 (95 % CI 0.57–0.72) *P* < 0.001

We used an ROC curve analysis to evaluate whether the NLR and PNI could predict DFS or OS (Fig. 2). The ROC curves identified an optimal NLR cutoff value of 2.5 for predicting DFS or OS in patients (DFS; AUC = 0.63, *P* = 0.001, OS; AUC = 0.62, *P* = 0.002). The ROC curves identified an optimal PNI cutoff value of 50 for predicting DFS or OS in patients (DFS; AUC = 0.62, *P* = 0.001, OS; AUC = 0.64, *P* < 0.001). We divided the patient population based on a NLR and PNI cutoff value of 2.5 and 50 for the patients. All the clinicopathological characteristics were comparable between patients according to their NLR or PNI statuses (Table 2). Our study showed that the NLR was associated with sex, smoking history, the CEA level, tumor size, and vascular invasion. On the other hand, the PNI was associated with sex, age, smoking history, tumor size, histological type, tumor differentiation, and vascular invasion. Interestingly, nodal metastasis was not associated with either the NLR or the PNI.

**Table 2:** Clinicopathological characteristics grouped by NLR and PNI status

	Cases	NLR			PNI		
		<2.5	≥2.5	*P* value	<50	≥50	*P* value
Sex							
Female	121	88	33	0.038	36	85	0.002
Male	213	131	82		100	113	
Age							
≦70	165	116	49	0.072	45	120	<0.001
>s70	169	103	66		91	78	
Smoking history							
Never	123	90	33	0.026	39	84	0.010
Smoker	211	129	82		97	114	
CEA							
Normal	231	165	66	0.001	87	144	0.089
High	103	54	49		49	54	
Tumor size							
T1	161	116	45	0.016	51	110	0.001
T2 + 3	173	103	70		85	88	
Nodal metastasis							
Negative	270	181	89	0.246	110	160	0.986
Positive	64	38	26		26	38	
Histological type							
Adenoca	231	159	72	0.060	81	150	0.002
Non-adenoca	103	60	43		55	48	
Tumor differentiation							
Well	118	85	33	0.066	39	79	0.035
Mod + por	216	134	82		97	119	
Vascular invasion							
Negative	190	139	51	0.001	68	122	0.035
Positive	144	80	64		68	76	
NLR				–			
<2.5	220	–	–		54	165	<0.001
≥2.5	114	–	–		82	33	
PNI							–
<50	136	54	82	<0.001	–	–	
≥50	198	165	33		–	–	

*NLR* neutrophil/lymphocyte ratio, *PNI* prognostic nutritional index, *adenoca* adenocarcinoma, *mod+por* moderate+poorly

### Surgical factors and recurrence grouped by NLR and PNI status

The surgical factors and recurrence were comparable between patients according to their NLR or PNI statuses (Table 3). In this study, no patient had received pneumonectomy because all patients who had gone pneumonectomy between 2007 and 2012 had received induction chemotherapy or chemoradiotherapy and excluded from this study. Significant associations of the NLR ≥2.5 or PNI <50 were observed with the rate of thoracotomy (*P* = 0.044) but not with the type of resection (*P* = 0.997). Significant associations of the NLR ≥2.5 or PNI <50 were observed with the rate of recurrence; however, significant associations were not observed with the first recurrence site (local or distant metastasis).

**Table 3:** Surgical factor and recurrence grouped by NLR and PNI status

Cases	NLR			PNI		
	<2.5	≥2.5	*P* value	<50	≥50	*P* value
Type of resection						
Segmentectomy	33	17	0.997	20	30	0.959
Lobectomy	186	98		135	196	
Surgical Approach						
VATS	142	64	0.044	74	132	0.005
Thoracotomy	77	51		62	66	
Recurrence						
Negative	173	77	0.016	90	160	0.003
Positive	46	38		46	38	
First recurrence site						
Local	15	12	0.154	11	16	0.193
Distant	24	19		27	16	
Both	7	7		8	6	

*NLR* neutrophil/lymphocyte ratio, *PNI* prognostic nutritional index, *VATS* video-assisted thoracic surgery

### Prognostic analysis

**Fig. 3:**
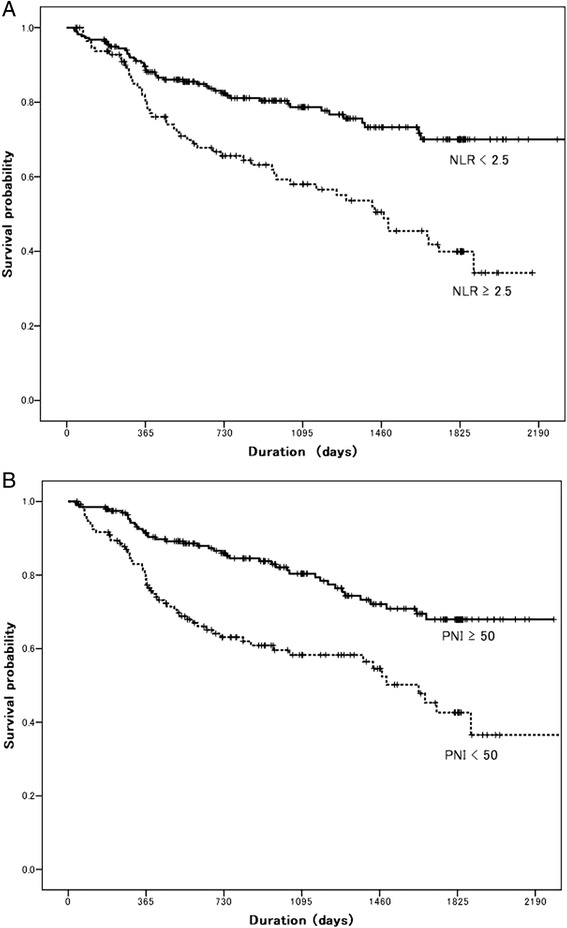
**a** Kaplan-Meier disease-free survival curve according to the NLR: log-rank *P* < 0.001. **b** Kaplan-Meier disease-free survival curve according to the PNI: log-rank *P* < 0.001

The 3-year DFS was 71.5 % in this study. Patients with NLR ≥2.5 had a significantly poorer survival outcome, compared with those with NLR < 2.5 (78.7 vs. 58.0 %, *P* < 0.001, according to the log-rank test; Fig. 3a). On the other hand, patients with PNI <50 had a significant poorer survival outcome, compared with those with PNI ≥50 (80.4 vs. 58.3 %, *P* < 0.001, according to the log-rank test; Fig. 3b). A univariate analysis showed that age, tumor size, nodal metastasis, tumor differentiation, the NLR, and the PNI were predictors of the DFS. A multivariate analysis was then performed using the Cox proportional hazards model. Using this model, we demonstrated that age (*P* = 0.004) and nodal metastasis (*P* < 0.001), tumor differentiation (*P* = 0.047), the NLR (*P* = 0.039), and the PNI (*P* = 0.007) were independent predictors of the DFS (Table 4). Overall survival (OS) data are immature, but a multivariate analysis suggested that age, nodal metastasis, NLR, and PNI were independent predictors of OS (Table 4).

**Table 4:** Multivariate analysis of factors predicting disease-free and overall survival

Disease-free survival						
	Univariate			Multivariate		
	HR	95 % CI	*P* value	HR	95 % CI	*P* value
Sex						
Male/female	1.70	1.08–2.69	0.023	1.37	0.85–2.19	0.187
Age						
>70/≦70	2.42	1.58–3.71	<0.001	1.94	1.24–3.04	0.004
Tumor size						
T2–3/T1	2.25	1.47–3.45	<0.001	1.14	0.74–1.76	0.560
Nodal metastasis						
Positive/negative	6.50	4.30–9.80	<0.001	6.42	4.16–9.89	<0.001
Histological type						
AD/non-AD	1.45	0.95–2.20	0.083	0.72	0.46–1.13	0.150
Tumor differentiation						
Mod+por/well	2.71	1.65–4.44	<0.001	1.69	1.01–2.83	0.047
NLR						
≥2.5/<2.5	2.09	1.40–3.12	<0.001	1.58	1.02–2.43	0.039
PNI						
<50/≥50	2.28	1.52–3.42	<0.001	1.88	1.19–2.97	0.007
Overall survival						
Sex						
Male/female	1.76	1.04–2.97	0.035	1.19	0.67–2.10	0.554
Age						
>70/≦70	3.54	2.07–6.05	<0.001	2.31	1.30–4.08	0.004
Tumor size						
T2-3/T1	2.60	1.56–4.31	<0.001	1.45	0.85–2.46	0.171
Nodal metastasis						
Positive/negative	4.21	2.62-6.74	<0.001	3.78	2.34–6.13	<0.001
Histological type						
AD/non-AD	1.20	1.05–1.36	0.006	1.07	0.92–1.24	0.373
Tumor differentiation						
Mod+por/well	2.81	1.59–4.99	<0.001	1.60	0.85–3.02	0.143
NLR						
≥2.5/<2.5	1.81	1.14–2.87	0.012	1.60	1.04–2.54	0.048
PNI						
<50/≥50	3.37	2.07–5.48	<0.001	2.40	1.39–4.14	0.002

*NLR* neutrophil/lymphocyte ratio, *PNI* prognostic nutritional index, *AD* adenocarcinoma, *mod+por* moderate+poorly

## Discussion

To our knowledge, this is one of the largest studies to evaluate the value of NLR and/or PNI in predicting the outcome of patients with NSCLC. In addition, this is the first study to show a correlation between the NLR and the PNI, as well as the prognostic impact of both the NLR and the PNI among patients with NSCLC. Our study demonstrated a positive correlation between the NLR and the PNI, and a high NLR (≥2.5 vs. <2.5, *P* = 0.039) and a low PNI (<50 vs. ≥50, *P* = 0.007) were significantly associated with a poor survival in the multivariate analysis.

The NLR is an inexpensive, reproducible, and widely available blood test. The preoperative NLR reflects the inflammation status and has been found to be an important indicator of an adverse prognosis among patients with various cancers. In resected NSCLC, Sarraf et al. reported that an increased preoperative NLR was associated with a higher stage and remained an independent predictor of overall survival [8]. Accumulating evidence supports the involvement of systemic inflammation in cancer progression. Cancer-related inflammation includes the presence of inflammatory cells and mediators in the tumor microenvironment. Neutrophils are a major constituent of cancer-related inflammation as well as the host defense against bacterial infection. Tumor-associated neutrophils can function as immunosuppressive cells in the presence of tumors [21]. On the other hand, circulating neutrophils have been shown to produce cytokines that contribute to cancer progression, and an elevated number of neutrophils suppress lymphokine-activated killer cells, thereby increasing the propensity for metastasis [22, 23]. Thus, the systemic inflammatory response has been associated with a poorer prognosis among patients with various cancers including NSCLC.

Patients with an elevated NLR exhibit a relative lymphocyte ratio and may have a poorer lymphocyte-mediated immune response to tumors, thereby increasing tumor progression and worsening the prognosis [24]. In this regard, the NLR seems to be a potential indicator mirroring both host immunity and neutrophil-dependent inflammation, which is associated with the clinical outcome. Particularly, in patients who had undergone a complete resection of the main tumor, the host cell-mediated immunity continues to exert important effects on the destruction of residual tumor cells and micrometastasis. Accordingly, a higher NLR was found to be associated with a poor DFS.

Assessment and support of the nutritional status should be considered a valuable component of the overall oncological strategy [25]. The PNI was reported by Smale et al. to predict the risk of operative morbidity and mortality after gastrointestinal surgery [26]. However, their method for calculating the PNI was too difficult to use routinely. In contrast, the simplified PNI reported by Onodera et al. was based on only two laboratory parameters, the albumin level and the lymphocyte count, which can be easily measured and are routinely used in clinical practice [13]. The PNI was initially designed to assess the nutritional and immunological statuses of patients undergoing gastrointestinal surgery. In 2011, Proctor et al. reported that Onodera’s PNI could predict the outcome of cancer patients, regardless of the primary site of origin [19]. However, to our knowledge, few studies have examined the PNI in patients with NSCLC. In patients with lung cancer, a smoking habit is frequently associated with malnutrition. Chronic obstructive pulmonary disease (COPD) and aging are independent and probably concurrent conditions leading to malnutrition [27]. Shinozawa et al. reported that cigarette smoking and an advanced age were well-known risk factors for COPD, and a lower lung function was independently and linearly associated with lower blood markers for nutritional status and anemia [28]. In our study, the mean PNI was significantly lower among older patients, former and current smokers, and those with squamous cell carcinoma. In patients with lung cancer, the PNI is probably associated not only with the immunological status but also with cigarette smoking.

This study has several limitations that should be considered when interpreting the results. The retrospective study design was a major limitation of the present study. Minor limitations included insufficient evidences of the validity of the cutoff values for the NLR and the PNI. Regarding the NLR, a fixed cutoff value has not yet been established, and various values have been used in previous reports. Because many studies did not describe the method used to select the NLR cutoff, we used a cutoff value based on a previous study. Regarding the PNI, fixed cutoff value has also not yet been established, and various values have been used in previous reports.

## Conclusions

In conclusion, a significant inverse correlation between the NLR and the PNI and a high NLR and a low PNI were significantly associated with a poor survival among patients who had undergone a complete resection for NSCLC. NLR and PNI may help in assessing the treatment strategy such as adjuvant therapy and the examination interval after surgery.

## Abbreviations

NLR: neutrophil/lymphocyte ratio
NSCLC: non-small cell lung cancer
PNI: prognostic nutritional index
